# Angiogenesis Inhibitors in NSCLC

**DOI:** 10.3390/ijms18102021

**Published:** 2017-09-21

**Authors:** Anna Manzo, Agnese Montanino, Guido Carillio, Raffaele Costanzo, Claudia Sandomenico, Nicola Normanno, Maria Carmela Piccirillo, Gennaro Daniele, Francesco Perrone, Gaetano Rocco, Alessandro Morabito

**Affiliations:** 1 Thoracic Medical Oncology, Istituto Nazionale Tumori, “Fondazione G.Pascale”—IRCCS, 80131 Napoli, Italy; anna_manzo1@hotmail.it (A.M.); agnese.montanino@libero.it (A.M.); rc71@libero.it (R.C.); csando@libero.it (C.S.); 2 Department of Oncology and Hematology, Azienda Ospedaliera Pugliese-Ciaccio, 88100 Catanzaro, Italy; guidocaro@alice.it; 3 Cellular Biology and Biotherapy, Research Department, Istituto Nazionale Tumori “Fondazione G.Pascale”—IRCCS, Napoli 80131, Italy; n.normanno@istitutotumori.na.it; 4 Clinical Trials Unit, Istituto Nazionale Tumori, “Fondazione G.Pascale”—IRCCS, 80131 Napoli, Italy; marilinapiccirillo@yahoo.it (M.C.P.); g.daniele@istitutotumori.na.it (G.D.); f.perrone@istitutotumori.na.it (F.P.); 5 Thoracic Surgery, Istituto Nazionale Tumori, “Fondazione G.Pascale”—IRCCS, 80131 Napoli, Italy; g.rocco@istitutotumori.na.it

**Keywords:** angiogenesis, vascular endothelial growth factor (VEGF), bevacizumab, nintedanib, ramucirumab, VEGF trap

## Abstract

Angiogenesis is a complex biological process that plays a relevant role in sustaining the microenvironment, growth, and metastatic potential of several tumors, including non-small cell lung cancer (NSCLC). Bevacizumab was the first angiogenesis inhibitor approved for the treatment of patients with advanced NSCLC in combination with chemotherapy; however, it was limited to patients with non-squamous histology and first-line setting. Approval was based on the results of two phase III trials (ECOG4599 and AVAIL) that demonstrated an improvement of about two months in progression-free survival (PFS) in both trials, and in the ECOG4599 trial, an improvement in overall survival (OS) also. Afterwards, other antiangiogenic agents, including sunitinib, sorafenib, and vandetanib have been unsuccessfully tested in first and successive lines. Recently, two new antiangiogenic agents (ramucirumab and nintedanib) produced a significant survival benefit in second-line setting. In the REVEL study, ramucirumab plus docetaxel prolonged the median OS of patients with any histology NSCLC when compared with docetaxel alone (10.4 versus 9.1 months, hazard ratio (HR) 0.857, *p* = 0.0235). In the LUME-Lung 1 study, nintedanib plus docetaxel prolonged the median PFS of patients with any tumor histology (*p* = 0.0019), and improved OS (12.6 versus 10.3 months) in patients with adenocarcinoma. As a result, it became a new option for the second-line treatment of patients with advanced NSCLC and adenocarcinoma histology. Identifying predictive biomarkers to optimize the benefit of antiangiogenic drugs remains an ongoing challenge.

## 1. Introduction

Lung cancer is the leading cause of cancer deaths worldwide [1]. However, a better understanding of the biology of this cancer in recent years has led to the development of therapies that have changed the treatment of non-small cell lung cancer (NSCLC), in particular when molecular alterations are present. In the absence of the molecular alterations that occur in a limited subgroup of patients (approximately 20% of Caucasian population), the majority of patients with NSCLC are candidates for first-line chemotherapy with platinum-based doublets [2]. In these patients, targeting the angiogenesis pathways represents an alternative and attractive strategy. Angiogenesis is a complex process that plays a central role in sustaining cancer microenvironment, tumor growth, and metastatic dissemination [3]; it is involved in a delicate balance both proangiogenic and antiangiogenic molecules. The vascular endothelial growth factor (VEGF) family, including different VEGF isoforms generated through the alternative splicing of VEGF mRNA and placenta-derived growth factor (PDGF), has a key role in this process [4]. Among the isoforms, VEGF-A (VEGF) is the main mediator of physiological and tumor-associated angiogenesis [5]. There are three associated transmembrane receptors for VEGF family ligands, Flt-1 (VEGFR-1), KDR (VEGFR-2), and Flt-4 (VEGFR-3), which are related to the platelet-derived growth factor receptor (PDGFR) family [6]. Each receptor has intrinsic tyrosine kinase activity that is stimulated after ligand binding and receptor dimerization, and is crucial for transmission of a cytoplasmic signaling response. The result of this cascade is the creation of new blood vessels that are different from normal vessels; with anarchic organization and heterogeneity of the blood flow, these vessels provide oxygen and nutrients, promote tumor growth, and allow tumor cells to escape into the circulation, thus leading to metastasis.

**Figure 1:**
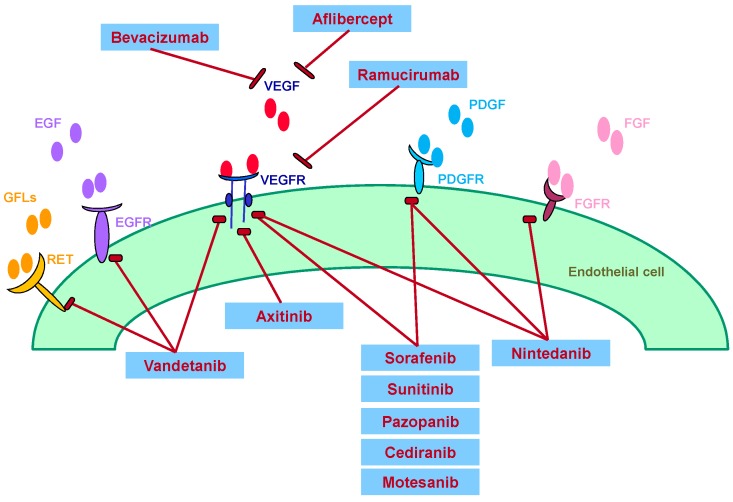
Mechanisms of action of angiogenesis inhibitors targeting VEGF, EGF, PDGF, FGF, RET and their receptors for suppressing angiogenesis. VEGF: vascular endothelial growth factor; EGF: epidermal growth factor; PDGF: placenta-derived growth factor; FGF: fibroblast growth factor; RET: *REarranged during Transfection* (gene).

The well-established role of VEGF in promoting tumor angiogenesis and the pathogenesis of cancers has led to the rational design and development of agents that selectively target this pathway. Angiogenesis inhibitors used to treat NSCLC include monoclonal antibodies, small molecule VEGF receptor-tyrosine kinase inhibitors (VEGFR-TKIs), and VEGF Trap (Figure 1) [7].

## 2. Bevacizumab

Bevacizumab, a humanized monoclonal antibody directed against VEGF, was the first angiogenesis inhibitor approved for first-line treatment of patients with lung adenocarcinoma, based on the results of two phase III trials [8,9,10]. The first trial, ECOG4599, compared bevacizumab plus carboplatin and paclitaxel followed by bevacizumab maintenance with carboplatin and paclitaxel in 878 patients with non-squamous NSCLC. The trial demonstrated a statistically significant benefit in favor of the combination of chemotherapy plus bevacizumab in terms of both overall survival (OS) (12.3 versus 10.3 months, hazard ratio (HR) 0.79, 95% CI: 0.67–0.92, *p* = 0.003) and progression-free survival (PFS) (6.2 versus 4.5 months, HR 0.66, 95% CI: 0.57–0.77, *p* < 0.001) [8] (Table 1). Moreover, a four-month survival benefit with the addition of bevacizumab was observed in the subgroup of patients with adenocarcinoma histotype (14.2 versus 10.3 months, HR 0.69; 95% CI: 0.58–0.83) [9]. The AVAIL trial compared bevacizumab with a non-taxane-based chemotherapy with a non-taxane-based chemotherapy alone in 1043 patients with non-squamous NSCLC in the first-line setting, and demonstrated that bevacizumab significantly extended PFS and improved the response rate versus a placebo when added to cisplatin plus gemcitabine, but did not significantly extend OS (Table 1), probably because of a higher rate of post-study treatments than in the ECOG4599 trial [10]. Main adverse events (AEs) associated with bevacizumab included hypertension, proteinuria, bleeding, and neutropenia.

A meta-analysis, including these two trials and two phase 2 trials, and 2194 NSCLC patients overall, was conducted by Soria et al. to assess the efficacy (in terms of OS and PFS) and toxicity of bevacizumab used in combination with first-line platinum-based chemotherapy, compared with chemotherapy alone. The meta-analysis demonstrated a significant benefit in terms of both OS (HR 0.90, 95% CI: 0.81–0.99, *p* = 0.03) and PFS (HR 0.72, 95% CI: 0.66–0.79, *p* < 0.001). No unexpected toxicity was recorded [14]. Recently, another meta-analysis was conducted by Behera et al. to determine whether the benefit of adding bevacizumab in the first-line setting was restricted to a taxane-based or a non-taxane-based combination regimen [15]. From the analysis of 29 trials and 5890 patients (2767 and 3123 in the taxane and non-taxane group, respectively), the outcomes of the two combination regimens appeared to be similar. In particular, median OS was 14.4 versus 13.7 months (*p* = 0.5), median PFS was 6.93 versus 6.99 months (*p* = 0.61), and response rate was 41% versus 39% (*p* = 0.65) in the taxane and non-taxane group, respectively.

With the emerging role of pemetrexed in the treatment of non-squamous NSCLC, there was also interest in evaluating the combination of bevacizumab with pemetrexed (Table 1). In the AVAPERL trial, maintenance bevacizumab plus pemetrexed significantly prolonged PFS compared with bevacizumab alone (7.4 versus 3.7 months, HR 0.57, 95% CI: 0.44–0.75, *p* < 0.0001) without unexpected toxicities. The most common any grade AEs during the maintenance were nausea, hypertension, and asthenia in both arms. Severe hematologic toxicity was reported in the combination arm only (neutropenia 5.6%, anemia 3.2%) [11]. The Point Break study compared bevacizumab plus carboplatin and pemetrexed followed by continuation maintenance with bevacizumab plus pemetrexed (PemCBev) with standard bevacizumab plus carboplatin and paclitaxel, followed by bevacizumab maintenance (PacCBev) [12]. This trial did not meet its primary endpoint, showing no difference in OS, although PFS was significantly improved with PemCBev (6.0 vs. 5.6 months; HR 0.83, 95% CI: 0.71–0.96, *p* = 0.012). Median PFS for the maintenance population was 8.6 months for PemCBev, and 6.9 months for PacCBev. The median OS achieved in both arms (12.6 months with PemCBev and 13.4 months with PacCBev) was comparable to the median OS in the paclitaxel plus carboplatin plus bevacizumab arm in the ECOG4599 trial (12.3 months). Toxicity profiles differed between the two arms: significantly more grade 3 or 4 anemia (14.5% vs. 2.7%), thrombocytopenia (23.3% vs. 5.6%), and fatigue (10.9% vs. 5.0%) occurred with PemCBev; significantly more grade 3 or 4 neutropenia (40.6% vs. 25.8%), febrile neutropenia (4.1% vs. 1.4%), sensory neuropathy (4.1% vs. 0%), and alopecia (grade 1 or 2; 36.8% vs. 6.6%) occurred with PacCBev. The impact of the maintenance bevacizumab plus pemetrexed could be definitively clarified by the results of ongoing trials, such as the ECOG5508 trial, in which patients were randomly assigned to receive bevacizumab alone or bevacizumab plus pemetrexed or pemetrexed alone as maintenance [16].

An Italian randomized phase III trial (ERACLE) compared the quality of life (QoL) of patients with non-squamous tumor histology treated with bevacizumab plus carboplatin and paclitaxel followed by bevacizumab maintenance or cisplatin plus pemetrexed followed by pemetrexed maintenance [13]. The ERACLE study failed in demonstrating an advantage in QoL in one of the study arms. Moreover, no differences were reported for efficacy outcomes, although the trial was not powered for this purpose.

Interesting data come from the combination of bevacizumab with target therapy. In a double-blind, placebo-controlled, randomized phase 3 trial (BeTa), the addition of bevacizumab to erlotinib did not improve survival in 636 patients with recurrent or refractory NSCLC, after failure of first-line treatment [17]. However, a prolongation PFS was observed (3.4 versus 1.7 months, HR 0.60). A subsequent Japanese randomized phase II trial investigated the combination of erlotinib and bevacizumab in the first-line treatment of patients with activating epidermal growth factor receptor (EGFR) mutation-positive advanced NSCLC. In this trial, 154 patients were randomly assigned to receive erlotinib and bevacizumab (*n* = 77), or erlotinib alone (*n* = 77). Median PFS was 16.0 months with erlotinib plus bevacizumab, and 9.7 months with erlotinib alone (HR 0.54, 95% CI: 0.36–0.79; *p* = 0.0015). The most common grade 3 or worse adverse events were rash (25% with erlotinib plus bevacizumab vs. 19% with erlotinib alone), hypertension (60% vs. 10%), and proteinuria (8% vs. none). Serious adverse events occurred at a similar frequency in both groups (24% with erlotinib plus bevacizumab and 25% with erlotinib alone) [18]. BELIEF (bevacizumab and erlotinib in EGFR mutation-positive NSCLC), an European single-arm, phase 2 study of erlotinib plus bevacizumab, which enrolled 109 patients with *EGFR*-mutated NSCLC, confirmed the promising results of the Asian trial. Median PFS was 13.6 months, with a one-year PFS rate of 55.6%. Also, intriguing data emerged for patients with de novo T790 EGFR mutation: in this subgroup of 60 patients, the one-year PFS rate was 60.2%, and median PFS was 15.4 months [19]. Based on this evidence, a randomized, open-label, phase III trial combining first-line erlotinib plus bevacizumab in patients with advanced non-squamous NSCLC harboring EGFR mutations is ongoing [20].

Preclinical evidences in different tumor types suggest that when suspending antiangiogenic drugs, a “rebound” effect could appear, with an accelerated tumor progression. Therefore, continuing the antiangiogenic treatment beyond progression may be beneficial, as demonstrated in patients with metastatic colorectal cancer [21,22,23]. The efficacy and safety of continuing bevacizumab beyond progression after first-line treatment was evaluated in patients with advanced non-squamous NSCLC, in an open-label, randomized, phase IIIb trial [24]. The study did not meet the primary endpoint, showing no difference in OS, although efficacy data suggest a positive trend for continued bevacizumab plus standard of care (SOC) beyond progression compared with SOC alone [25]. Median OS was 11.9 months with bevacizumab versus 10.2 months for SOC alone (HR 0.84, 90% CI: 0.71–1.00; *p* = 0.1016; 387 OS events). OS rates were 10% higher in the bevacizumab arm versus SOC alone at six, 12, and 18 months. Overall response rate (ORR) and disease control rate (DCR) were slightly higher in the bevacizumab arm versus the SOC arm (ORR 9.7% vs. 6.7%; DCR 86.2% vs. 79.3%, respectively). No cumulative safety signals were identified. Grade ≥ 3 adverse events were reported in 78.2% of bevacizumab arm, and 61.6% of SOC arm.

Bevacizumab was also tested in combination with standard treatment for locally advanced and for completely resected early-stage NSCLC. Unfortunately, no positive results in terms of efficacy and safety have been observed for stage III lung cancer [26], and negative results have recently been reported in the adjuvant setting [27].

Despite various attempts in different settings, the use of bevacizumab with a platinum based chemotherapy has been demonstrated to improve OS and to be well tolerated in patients with advanced non-squamous NSCLC only in the first-line setting. However, the use of this combination is not without limits: in the absence of biomarkers identifying patients who might gain the most benefit from this combination, only clinical features can be used to minimize the toxicity of antiangiogenic drugs. Therefore, bevacizumab should be considered for selected patients eligible for antiangiogenic treatment: patients with no cardiovascular comorbidities, no tumor cavitation, no major vessel invasion, no previous hemoptysis, no recent thromboembolic disease, no severe or uncontrolled hypertension, and age preferably <75 years.

## 3. Sunitinib, Sorafenib, Vandetanib

To date, several phase II and III clinical trials have been conducted evaluating small-molecule tyrosine kinase inhibitors (TKIs) targeting the VEGF receptor (VEGFR) pathway. These drugs, including vandetanib, sorafenib, sunitinib, pazopanib, axitinib, cediranib, and motesanib, have been evaluated in combination with chemotherapy, in combination with erlotinib, or as single agents (Table 2).

Two randomized, phase III, double-blind, placebo-controlled, clinical trials evaluated the efficacy of the addition of vandetanib, a multikinases (VEGFR, EGFR, RET) inhibitor, to chemotherapy in patients with locally advanced or metastatic NSCLC: the ZODIAC and the ZEAL trial. The ZODIAC trial compared vandetanib 100 mg plus docetaxel versus docetaxel monotherapy as second-line treatment of 1391 patients [28]. The study met its primary objective of PFS prolongation with the addition of vandetanib to docetaxel vs. docetaxel (HR 0.79, 97.58% CI: 0.70–0.90; *p* < 0.0001). Median PFS was 4 months in the vandetanib arm vs. 3.2 months in the placebo arm. Significant advantages for vandetanib plus docetaxel were also seen for response rate (17% versus 10%, *p* < 0.001) and time to deterioration of symptoms (HR 0.78, *p* = 0.002; FACT-L Lung Cancer Subscale). However, no advantage in OS was shown with vandetanib plus docetaxel (HR 0.91, 97.52% CI: 0.78–1.07, *p* = 0.196). Among grade ≥3 adverse events, rash (9% vs. 1%), neutropenia (29% vs. 24%), leukopenia (14% vs. 11%), and febrile neutropenia (9% vs. 7%) were more common with vandetanib plus docetaxel than with placebo plus docetaxel. The ZEAL trial compared vandetanib (100 mg) plus pemetrexed with pemetrexed monotherapy in 534 patients as second-line treatment for advanced NSCLC [29]. There were statistically significant advantages for response rate (19% versus 8%, *p* < 0.001) and time to deterioration of symptoms (HR 0.71, *p* = 0.0052), but the study did not meet its primary end point of PFS (HR 0.86, 97.58% CI: 0.69–1.06, *p* = 0.108) and OS (HR 0.86, 97.54% CI: 0.65–1.13, *p* = 0.219) prolongation. Vandetanib increased the incidence of some adverse events, including rash, diarrhea, and hypertension.

Vandetanib as single agent (300 mg) was compared with gefitinib in a randomized double-blind phase II study in 168 pretreated NSCLC patients [39]. The study had a crossover design, to assess the activity of vandetanib also in patients who failed treatment with gefitinib. The first part of the study (before the crossover) met its primary end point: median PFS was 11 weeks for vandetanib and 8.1 weeks for gefitinib (HR 0.69, 95% CI: 0.50–0.96, *p* = 0.025). Partial responses were observed in seven patients (8%) receiving vandetanib and in one patient (1%) receiving gefitinib. The OS analysis showed no benefit for patients initially assigned to vandetanib compared with gefitinib (HR 1.19, 95% CI: 0.84–1.68, two-sided *p* = 0.34), but the crossover design might have confounded survival assessment. In particular, median OS was 6.1 months for patients treated with vandetanib followed by gefitinib, and 7.4 months for patients treated with gefitinib followed by vandetanib. The most common adverse events with both treatments were diarrhea, fatigue, nausea, and rash. Two further randomized, phase III, double-blind, trials investigated the efficacy of vandetanib as a single agent in NSCLC patients: the ZEST trial, which compared vandetanib with erlotinib in patients with locally advanced or metastatic NSCLC after failure of at least one prior anti-cancer therapy, and the ZEPHYR trial, which tested vandetanib versus placebo in patients with refractory NSCLC who failed chemotherapy and an anti-EGFR therapy. The ZEST trial did not meet its primary objective of demonstrating PFS prolongation with vandetanib 300 mg vs. erlotinib 150 mg in 1240 patients with previously treated advanced NSCLC (HR 0.98, 95% CI: 0.87–1.10, *p* = 0.721) [30]. Moreover, there was no difference in OS (HR 1.01, 95% CI: 0.89–1.16, *p* = 0.830), response rate, or time to deterioration of symptoms. The ZEPHYR trial, dedicated to heavily pretreated NSCLC patients, and randomized 2:1 to receive vandetanib 300 mg (*n* = 617) or placebo (*n* = 307), also did not meet its primary end point of showing superiority in OS [31]. Median OS was 8.5 vs. 7.8 months (HR 0.95, 95% CI: 0.81–1.11, *p* = 0.527). Significant advantages favoring vandetanib were seen for the secondary end points, such as PFS, ORR, and disease control rate at eight weeks, but not for time to deterioration of symptoms. Common adverse events occurring with a higher frequency in the vandetanib arm included diarrhea (46% vs. 11%), rash (42% vs. 11%), and hypertension (26% vs. 3%).

Negative results were observed with sorafenib in combination with chemotherapy in the first-line setting. A multicenter, randomized, placebo-controlled trial assessed the efficacy and safety of sorafenib in combination with carboplatin and paclitaxel as first line in patients with unresectable stage IIIB or IV NSCLC [32]. Nine hundred and twenty-six patients were randomly assigned to receive up to six cycles of carboplatin AUC6 and paclitaxel 200 mg/m^2^ on day 1 every 21 days, followed by either sorafenib 400 mg twice a day (*n* = 464, arm A) or placebo (*n* = 462, arm B) on days 2 to 19. Maintenance with sorafenib or placebo was planned after chemotherapy. Two hundred and twenty-three patients (24%) had squamous cell histology. Median OS, the primary end point, was 10.7 months in arm A, and 10.6 months in arm B (HR 1.15, 95% CI: 0.94–1.41, *p* = 0.915). The interim analysis concluded that the study was highly unlikely to meet its primary end point. Patients with squamous cell histology had greater mortality in arm A than in arm B (HR 1.85, 95% CI: 1.22–2.81). Main grade 3 or 4 sorafenib-related toxicities included rash (8.4%), hand-foot skin reaction (7.8%), and diarrhea (3.5%). An analogous phase III study tested the safety and efficacy of sorafenib plus cisplatin and gemcitabine as first line in patients with unresectable stage IIIB or IV NSCLC [33]. After considering data from previous studies, patients with squamous histology were withdrawn from the trial and excluded from analysis. Nine hundred and four patients were randomly assigned to daily sorafenib (400 mg twice a day) or matching placebo plus cisplatin 75 mg/ m^2^ on day 1 and gemcitabine 1250 mg/m^2^ on days 1 and 8 for up to six 21-day cycles. Median OS, the primary endpoint, was similar in the sorafenib and placebo groups (12.4 vs. 12.5 months; HR 0.98, *p* = 0.401). By investigator assessment, sorafenib improved median PFS (6.0 vs. 5.5 months; HR 0.83, *p* = 0.008) and time to progression (TTP) (6.1 versus 5.5 months, HR 0.73, *p* < 0.001). Grade 3 to 4 drug-related adverse events included hand–foot skin reaction (8.6% vs. 0.3%), fatigue (7.3% vs. 3.6%), rash (5.7% vs. 0.5%), and hypertension (4.2% vs. 1.8%). A phase III placebo-controlled study evaluated sorafenib monotherapy after at least two prior regimens in 703 patients [34]. Median OS, the primary endpoint, was similar in the sorafenib and placebo groups (8.2 vs. 8.3 months; HR 0.99, 95% CI: 0.84–1.17, *p* = 0.47). Median PFS (2.8 vs. 1.4 months; HR 0.61, 95% CI: 0.51–0.72, *p* < 0.0001) was significantly longer with sorafenib than with placebo. Among the 89 patients with EGFR mutations, OS (13.9 vs. 6.5 months, HR 0.48, 95% CI: 0.30–0.76, *p* = 0.002) and PFS (2.7 vs. 1.4 months, HR 0.27, 95% CI: 0.16–0.46, *p* < 0.001) were significantly longer with sorafenib than placebo. PFS was significantly longer with sorafenib than placebo also in patients with either wild-type or mutated KRAS, but OS was similar. Common drug-related adverse events were rash/desquamation, diarrhea, and fatigue.

Another TKI, sunitinib, was tested in patients with advanced NSCLC. A phase III study investigated the role of maintenance switch to sunitinib 37.5 mg daily or placebo after four cycles of first line cisplatin-based chemotherapy in patients with stable or responding disease [40]. Two hundred and ten patients were randomized; 22.4% received prior bevacizumab and 45.9% had adenocarcinoma. The study met its primary endpoint by demonstrating a significant improvement in median PFS with sunitinib maintenance (4.3 versus 2.8 months with placebo; HR 0.59, *p* = 0.0008). PFS was improved for both squamous (4.3 versus 2.4 months, HR 0.55, *p* = 0.02) and non-squamous histology (4.3 versus 2.8 months, HR 0.64, *p* = 0.02). However, OS was not different (11.2 months versus 11.2 months, HR 1.05, *p* = 0.77). Grade 3/4 toxicities occurring in > 5% of patients were anemia, neutropenia, thrombocytopenia, hypertension, fatigue, rash, and mucositis. A phase III trial investigated OS for sunitinib 37.5 mg plus erlotinib 150 mg daily versus placebo plus erlotinib in 960 patients with refractory NSCLC [35]. The primary end point was OS. Secondary end points included PFS, ORR, and safety. Median OS was 9 months for sunitinib plus erlotinib versus 8.5 months for erlotinib alone (HR 0.922, 95% CI: 0.797–1.067; one-sided stratified log-rank *p* = 0.1388). Median PFS was 3.6 months versus 2 months (HR 0.807, 95% CI: 0.695–0.937; one-sided stratified log-rank *p* = 0.0023), and ORR was 10.6% versus 6.9% (two-sided stratified log-rank *p* = 0.0471), respectively. Treatment-related grade 3 or higher toxicities, including rash/dermatitis, diarrhea, and asthenia/fatigue were more frequent in the sunitinib plus erlotinib arm.

Similarly, a phase II study randomized 192 patients with stage IIIB or IV NSCLC to receive erlotinib 150 mg and pazopanib 600 mg daily or erlotinib and placebo, following one to two lines of chemotherapy [41]. The combination regimen obtained a significant improvement of PFS (2.6 versus 1.8 months, HR 0.59, 95% CI: 0.43–0.83, *p* = 0.0016), but no advantage in OS (6.8 versus 6.7 months, HR 1.1, 95% CI: 0.77–1.55, *p* = 0.61). Common severe toxicities were diarrhea (19% vs. 9%), fatigue (20% vs. 14%), and proteinuria (5% vs. 0%).

Axitinib, a potent VEGFR1-2-3 TKI, was tested, at the dose of 5 mg twice daily, in combination with first line cisplatin 75 mg/m^2^ and pemetrexed 500 mg/ m^2^ vs. chemotherapy alone, in a phase II study enrolling 170 patients [42]. Neither PFS (8 versus 7.1 months, HR 0.89, *p* = 0.36) or OS (17 versus 15.9 months) resulted significantly better with axitinib. ORR was 45.5% in the combination arm vs. 26.3% with chemotherapy alone. Gastrointestinal disorders, hypertension, and fatigue were the most common non-hematological grade ≥ 3 toxicities. Another phase II study compared axitinib 5 mg twice daily or bevacizumab 15 mg/kg every 21 days combined with first-line carboplatin AUC6 and paclitaxel 200 mg/m^2^ in 118 patients with stage IIIB/IV non-squamous NSCLC [43]. The trial was discontinued after an interim analysis. Median PFS was 5.7 versus. 6.1 months (HR 1.09, 95% CI: 0.68–1.76, *p* = 0.64) for axitinib and bevacizumab, respectively; median OS was 10.6 versus 13.3 months (HR 1.12, 95% CI: 0.74–1.69, *p* = 0.70) and ORR was 29.3% (18.1-42.7) versus 43.3% (30.6–56.8), respectively. The most common grade 3/4 adverse events included neutropenia (28% vs. 20%), fatigue (14% vs. 7%), and hypertension (14% vs. 5%).

Cediranib, a VEGFR1-2-3 TKI, was tested, at the dose of 20 mg daily, in a phase III study in combination with standard first-line carboplatin AUC6 and paclitaxel 200 mg/m^2^ vs. chemotherapy alone [36]. The trial was stopped at an interim analysis for futility. Analyses were performed on the 306 enrolled patients out of the 750 planned. The addition of cediranib increased response rate (RR 52% versus 34%, *p* = 0.001), but did not significantly improve PFS (HR 0.91, 95% CI: 0.71–1.18, *p* = 0.49) or OS (HR 0.94, 95% CI: 0.69–1.30, *p* = 0.72). Cediranib and chemotherapy caused more grade 3 hypertension, diarrhea, and anorexia than chemotherapy alone.

Finally, multikinases (VEGFR1-2-3, PDGFR and KIT) inhibitor motesanib 125 mg daily was tested in patients with non-squamous stage IIIB/IV NSCLC in combination with first-line carboplatin AUC6 and paclitaxel 200 mg/m^2^ vs. chemotherapy alone [37]. One thousand and ninety patients were enrolled, 890 of whom had adenocarcinoma. Median OS, the primary end point, was not improved with motesanib in the overall population (13 versus 11 months; HR 0.90, 95% CI: 0.78–1.04, *p* = 0.14) or in the adenocarcinoma subset (13.5 versus 11 months; HR 0.88, 95% CI: 0.75–1.03, *p* = 0.11). Median PFS was 5.6 months versus 5.4 months (*p* < 0.001); ORR was 40% versus 26% (*p* < 0.001). The incidence of grade ≥ 3 toxicities was higher with motesanib treatment.

Unfortunately, although the role of the inhibition of angiogenesis signaling pathways has been widely validated, all these small molecule TKIs have failed to demonstrate meaningful improvement in OS in the context of phase III clinical trials, showing instead generally unfavorable toxicity profiles.

## 4. VEGF Trap: Aflibercept

Aflibercept is a soluble decoy receptor that acts like a "VEGF trap" binding circulating VEGF-A, VEGF-B, and placental growth factor (PIGF) with a greater affinity than the human native receptors. An open-label phase II trial explored the activity of intravenous aflibercept, 4 mg/kg every 14 days alone, in 98 patients with platinum- and erlotinib-resistant lung adenocarcinoma [44]. ORR, the primary end point, was only 2%. PFS was 2.7 months and OS 6.2 months. Six- and twelve-month survival rates were 54% and 29%, respectively. Common grade 3/4 toxicities included hypertension (23%), dyspnea (21%), and proteinuria (10%). A case of reversible posterior leukoencephalopathy was reported.

Another single arm phase II study evaluated the activity and safety of first-line aflibercept 6 mg/kg every 21 days in combination with cisplatin and pemetrexed for up to six cycles in patients with non-squamous NSCLC [45]. Aflibercept maintenance was planned until disease progression or intolerance. The study was prematurely closed due to some cases of reversible posterior leukoencephalopathy syndrome. Among 38 evaluable patients, ORR and PFS, the co-primary end points, were 26% and 5 months, respectively. The most common treatment-emergent adverse events were nausea (69%) and fatigue (67%), with frequent grade 3 or 4 hypertension (36%).

A double-blind, placebo-controlled phase III trial assigned 913 patients with advanced non-squamous NSCLC to receive aflibercept 6 mg/kg or placebo every 21 days in combination with docetaxel 75 mg/m^2^ after platinum failure [38]. 12.3% of patients had received prior bevacizumab. Aflibercept did not improve OS, the primary endpoint (10.1 months versus. 10.4 months with aflibercept and placebo, respectively; HR 1.01, 95% CI: 0.87–1.17, *p* = 0.90). However, PFS was 5.2 and 4.1 months (HR 0.82, 95% CI: 0.72–0.94, *p* = 0.0035), and ORR 23.3% versus 8.9% (*p* < 0.001), with aflibercept and placebo, respectively. Adverse events occurring more frequently in the aflibercept arm were neutropenia (28% vs. 21.1%), fatigue (11.1% vs. 4.2%), stomatitis (8.8% vs. 0.7%), and hypertension (7.3% vs. 0.9%).

Even aflibercept in the phase III trial did not show any benefit in OS, with a significant increase of AEs in patients treated with VEGF trap, and moreover, several cases of reversible posterior leukoencephalopathy were reported.

## 5. Nintedanib

Nintedanib is a novel triple angiokinase inhibitor targeting VEGFR1–3, platelet-derived growth factor receptor (PDGFR) α and β, fibroblast growth factor receptors (FGFR) 1–3, and, in addition, is also an inhibitor of the Src family, RET, and FLT3 [46,47]. Two randomized phase III clinical trials have evaluated the efficacy of nintedanib in patients with advanced NSCLC, in the second-line setting (Table 3). In the large randomized multicenter phase III trial LUME-Lung1, nintedanib in combination with docetaxel demonstrated clinically meaningful benefit in patients who progressed after a first-line chemotherapy [48]. The primary endpoint was PFS by central independent review and the secondary endpoint was OS, in a prespecified stepwise order; additional secondary endpoints included investigator-assessed PFS, tumor response by central review and investigator assessment, safety, and patient-reported quality of life (QoL). Patients were randomized in a 1:1 ratio to receive nintedanib 200 mg b.i.d. (bis in die) plus standard docetaxel 75 mg/mq, or placebo plus standard docetaxel. The addition of nintedanib to docetaxel significantly improved PFS in the overall population (median 3.4 months versus 2.7 months; HR 0.79, 95% CI: 0.68–0.92, *p* = 0.0019). The benefit in PFS was consistent regardless of gender, age, ethnicity, or performance status (PS). The median OS was significantly improved in the docetaxel plus nintedanib group in the first predefined population of patients with adenocarcinoma histology progressed within 9 months after the start of first-line chemotherapy (median OS increased from 7.9 to 10.9 months; HR 0.75, *p* = 0.0073). Similar results were observed in the second predefined population of patients with adenocarcinoma histology (12.6 months with nintedanib versus 10.3 months with placebo; HR 0.83, 95% CI: 0.70–0.99, *p* = 0.00359). An exploratory analysis was conducted in patients refractory to first-line chemotherapy: in this group of poor prognosis patients, an advantage of more than 3 months was observed with the addition of nintedanib to docetaxel compared with docetaxel alone (9.8 versus 6.3 months, HR 0.62, *p* = 0.0246). There was no difference in OS in the overall study population (median 10.1 months versus 9.1 months; HR 0.94, 95% CI: 0.83–1.05, *p* = 0.2720). Adverse events that were more common in the docetaxel plus nintedanib arm than the docetaxel plus placebo arm were: diarrhea (all grades: 42.3% vs. 21.8%; grade ≥ 3 6.6% vs. 2.6%), increases in alanine aminotransferase (all grades, 28.5% vs. 8.4%; grade ≥ 3 7.8% vs. 0.9%), nausea (all grades, 24.2% vs. 18.0%; grade ≥ 3, 0.8% vs. 0.9%), increases in aspartate aminotransferase (all grades, 22.5% vs. 6.6%; grade ≥ 3, 3.4% vs. 0.5%), decreased appetite (all grades, 22.2% vs. 15.6%; grade ≥ 3, 1.4% vs. 1.2%), and vomiting (all grades 16.9% vs. 9.3%; grade ≥ 3, 0.8% vs. 0.5%). There was no statistically significant increase in the incidence of bleeding and hypertension events by the addition of nintedanib. On the basis of the positive results of LUME-Lung 1 study, nintedanib has been approved in combination with docetaxel for the second-line treatment of patients with advanced lung adenocarcinoma.

The LUME-Lung 2 multicenter, randomized, double-blinded phase III trial investigated the efficacy and safety of nintedanib in combination with pemetrexed versus placebo plus pemetrexed in patients with locally advanced or metastatic non-squamous NSCLC with progression after chemotherapy [49]. The primary endpoint was centrally reviewed PFS; secondary endpoints were OS, investigator-assessed PFS, ORR, safety, and QoL. Patients were randomized in a 1:1 ratio to nintedanib 200 mg b.i.d. plus pemetrexed 500 mg/mq or placebo plus pemetrexed. Continuation of treatment until progression of disease or unacceptable toxicity was allowed in both arms. The primary endpoint was met even though the study was stopped prematurely, due to a planned interim analysis of investigator-assessed PFS. Intention to treat (ITT) analysis showed that treatment with nintedanib plus pemetrexed significantly prolonged PFS compared with placebo plus pemetrexed (4.4 vs. 3.6 months; HR 0.83, 95% CI: 0.7–0.99, *p* = 0.04). Disease control rate was also significantly increased in the nintedanib arm (61 vs. 53%, odds ratio 1.37, *p* = 0.039). No difference in OS was seen between the arms (HR 1.03). There was no increase in serious adverse events in the nintedanib arm, and there was no difference between the arms in the incidence of grade 3 or higher hypertension, bleeding, thrombosis, mucositis, or neuropathy. However, diarrhea that was not severe and reversible elevated liver enzymes were more common with nintedanib.

Until recently, the use of antiangiogenic TKI therapy in the treatment of NSCLC has been decidedly an unsuccessful strategy. Instead, nintedanib demonstrated clinically meaningful benefits, showing an improvement of two months in OS, with manageable AEs in patients with advanced non-squamous NSCLC who progressed after first-line chemotherapy, and in the early progressors (within 9 months after start of chemotherapy) in particular. On these bases, the combination of docetaxel and nintedanib can be considered a new option for the second-line treatment for patients with advanced NSCLC with adenocarcinoma histology.

## 6. Ramucirumab

Ramucirumab is a fully human immunoglobulin G1 that selectively binds with high affinity to the extracellular domain of the VEGF receptor-2 (VEGFR-2), which blocks the interaction of VEGFR-2 and VEGF ligands, thus inhibiting their signaling pathways and the consequential endothelial proliferation and migration [51]. It is the second monoclonal antibody directed against VEGF approved for treatment of patients with NSCLC. In a recent phase III randomized trial, REVEL, ramucirumab was tested in combination with docetaxel as second-line treatment of advanced NSCLC, significantly improving OS compared with placebo and docetaxel [50]. In this trial, 1253 patients with squamous or non-squamous NSCLC who had progressed during or after a first-line platinum-based chemotherapy were randomly allocated (1:1) to docetaxel plus ramucirumab (experimental arm, *n* = 628), or placebo (control arm, *n* = 625). Median OS was 10.5 months with ramucirumab plus docetaxel, and 9.1 months with placebo plus docetaxel (HR 0.86, 95% CI: 0.75–0.98; *p* = 0.023). Median PFS was 4.5 months with ramucirumab, and 3.0 months with placebo (HR 0.76, 95% CI: 0.68–0.86; *p* < 0.0001). The adverse events observed in the experimental arm (neutropenia, leucopenia, fatigue, and hypertension) were easily manageable with dose adjustments and supportive care. This trial led to the FDA (Food and Drug Administration) and EMA (European Medicines Agency) approval of ramucirumab in combination with docetaxel as second-line treatment of NSCLC patients (Table 3).

Ramucirumab is currently in clinical development in the first-line setting. A phase II single-arm study investigating the combination of ramucirumab, carboplatin, and paclitaxel every 3 weeks followed by maintenance with ramucirumab alone, demonstrated a promising PFS (6-month PFS rate 59.0%), an ORR of 55%, and a disease control rate (DCR) of 90% [52]. A randomized phase II trial was conducted to compare ramucirumab in combination with cisplatin/carboplatin and pemetrexed every 3 weeks, followed by maintenance ramucirumab and pemetrexed (experimental arm), with cisplatin/carboplatin and pemetrexed every 3 weeks, followed by maintenance pemetrexed (control arm) in a non-squamous population. PFS (the primary endpoint) was not significantly longer (7.2 versus. 5.6 months, with and without ramucirumab, respectively; HR 0.75, 95% CI: 0.55–1.03, *p* = 0.132), whereas a difference in DCR was reported in the experimental arm with ramucirumab (86% versus 70%, *p* = 0.031) [53].

Ramucirumab reported a significantly longer OS in both squamous and non-squamous tumors in the phase III Revel trial, which indicates its potential to become a new important option for second-line treatment of advanced NSCLC patients, with any histology, in a context where there was only docetaxel until recently. Given the peculiar AEs and overall modest but significant benefits of antiangiogenetic agents, patient selection remains crucial, and is mandatory for the identification of predictive biomarkers.

## 7. Discussion and Conclusions

Angiogenesis is crucial for tumor growth and metastatic dissemination, and VEGF has a fundamental role in this process. Therefore, targeting VEGF could theoretically result in a substantial advantage for the treatment of several cancers, including lung cancer. For patients with advanced NSCLC and non-squamous histology, the role of bevacizumab combined with chemotherapy in the first-line setting and of nintedanib combined with docetaxel in the second-line setting are now well established. The efficacy of ramucirumab combined with docetaxel in the second-line setting of patients with advanced NSCLC and any tumor histology has also been demonstrated (Table 4).

However, there are relevant open questions that need to be addressed regarding: the identification of predictive molecular biomarkers, the antitumor activity of angiogenesis inhibitors such as adjuvant or neoadjuvant in other settings, the incorporation of antiangiogenic treatments with the newest strategies such as immunotherapy, the combination of antiangiogenic drugs, and TKIs.

Identifying molecular biomarkers that can predict responses to angiogenesis inhibitors remains an important goal to optimize the clinical benefit of these agents. Unfortunately, to date, there are no validated biomarkers that predict the response to bevacizumab, ramucirumab, or nintedanib. Measurements of several biomarkers (VEGF, bFGF, ICAM, E-selectin) at baseline and at week 7 were conducted in a prospective correlative study within the E4599 trial [54]. In this study, baseline VEGF levels resulted in predictive responses to bevacizumab, but not prognostic. Patients with high baseline levels of plasma VEGF (>35.7 pg/mL) showed an increased probability of responding to the combination of bevacizumab with paclitaxel-carboplatin compared with chemotherapy alone, while those with low baseline VEGF levels had a similar response rate to both treatments. Moreover, patients treated with bevacizumab with low baseline ICAM levels had a 53% reduction in PFS hazard rate. These data could indicate that patients with lower tumor burden are those most likely to benefit from bevacizumab-based therapy. A phase II study to examine the value of *FGFR1* gene amplification as a predictor of nintedanib efficacy in patients with squamous cell NSCLC is currently ongoing (ClinicalTrials.gov: NCT01948141). 

The E1505 study evaluated the addition of bevacizumab for one year to adjuvant chemotherapy, in 1501 patients with early stage resected NSCLC [27]. The trial was stopped early for futility. No difference in overall survival (HR 0.99, *p* = 0.90), the primary endpoint of the study, nor in disease-free survival (HR 1.00, *p* = 0.95), were observed. Moreover, the addition of bevacizumab significantly increased neutropenia, hypertension, and overall grade 3–5 toxicities. The perioperative activity of bevacizumab was assessed in 50 patients with operable stage IB-IIIA non-squamous NSCLC in a phase II trial [55]. The primary endpoint, the rate of downstaging among patients treated with neoadjuvant docetaxel–cisplatin–bevacizumab combination therapy, was not met, with downstaging in 38% of cases. Furthermore, downstaging was not associated with improved OS following resection (3-year OS rate: 70% vs. 56% in the groups with and without downstaging, *p* = 0.24).

There is growing evidence showing relationships between angiogenesis and the immune system. In particular, proangiogenesis factors may have immunosuppressive activity, and it has been suggested that antiangiogenic agents can stimulate the immune system, while immunotherapies can also be antiangiogenic. Therefore, combining immunotherapy with antiangiogenic treatment may have a synergistic effect that enhances the efficacy of both treatments [56]. A phase I study evaluated the safety and preliminary activity of switching to nivolumab maintenance therapy, as monotherapy or combined with bevacizumab, in patients with advanced NSCLC who did not progress on first-line platinum-based chemotherapy [57]. No treatment-related grade 4 adverse events, and a low frequency of grade 3 adverse events, were reported. Median PFS was 37.1 weeks with nivolumab plus bevacizumab, while it was 16 and 21.4 weeks with nivolumab monotherapy in patients with squamous and non-squamous histology, respectively. Another phase I study evaluated the combination of ramucirumab and pembrolizumab in patients with advanced NSCLC, gastric adenocarcinoma, or urothelial carcinoma in progression after prior systemic therapy. Preliminary results from the dose-limiting toxicity (DLT) part of the study showed no unexpected safety concerns. No DLTs were reported in patients with NSCLC [58]. 

The combination of bevacizumab and TKIs has been explored in different settings of patients with advanced NSCLC, because they target different tumor growth pathways (angiogenesis and EGFR activity, respectively), with little overlap in their toxic-effect profiles. In the BeTa trial, in the second-line setting, the addition of bevacizumab to erlotinib did not improve survival of NSCLC patients, but significantly prolonged PFS. Also, in the subsequent Japanese trial, the combination of bevacizumab and erlotinib highly significantly prolonged PFS with a HR 0.54. Possible explanations of this positive result could be the improved drug delivery due to bevacizumab-related changes in tumor vessel physiology, resulting in increased intratumoral uptake of drugs or the effective blocking of angiogenesis signaling via the VEGF receptor and EGFR signaling pathways, which is thought to promote tumor growth. The BEVERLY randomized phase III trial that is currently ongoing will verify the efficacy of the combination of erlotinib and bevacizumab in Caucasian patients with advanced NSCLC and activating EGFR mutations (Clinicaltrial.gov ID: NCT02633189).

In conclusion, in the first-line setting, the addition of bevacizumab to chemotherapy prolonged the overall survival of patients with advanced non-squamous NSCLC, and it should be considered among treatment options for these patients. In the second line, the addition of nintedanib or ramucirumab to docetaxel prolonged overall survival in patients with non-squamous or any histology, respectively, and they should be considered in particular for patients who are refractory or with early progression (within 9 months) after first-line chemotherapy. The search of predictive factors of response to antiangiogenic inhibitors remains an important issue of clinical research.

## Figures and Tables

**Table 1:** Randomized phase III clinical studies with bevacizumab in NSCLC.

Author and Publication Year	Trial	Setting	Pts	Systemic Treatment	Results
Sandler, 2006 [8]	E4599	1st line Advanced NSCLC	878	Bevacizumab + carboplatin/paclitaxel vs. carboplatin/paclitaxe	OS: 12.3 vs. 10.3 months HR 0.79, *p* = 0.003 PFS: 6.2 vs. 4.5 months HR 0.66, *p* < 0.001
Reck, 2009 [10]	AVAIL	1st line Advanced NSCLC	1043	Bevacizumab 7,5 + cisplatin/gemcitabine vs. bevacizumab15 + cisplatin/gemcitabine vs. cisplatin/gemcitabine	PFS Beva7.5: 6.8 vs. 6.2 HR 0.75, *p* = 0.003 Beva 15: 6.6 months HR 0.82, *p* = 0.03
Barlesi, 2013 [11]	AVAPERL	Maintenance Advanced NSCLC	253	Bevacizumab + pemetrexed vs. bevacizumab, maintenance	PFS: 7.4 vs. 3.7 months HR 0.57, *p* < 0.0001
Patel, 2009 [12]	POINT BREAK	Maintenance Advanced NSCLC	939	Pemetrexed + carboplatin + bevacizumab followed by maintenance pemetrexed + bevacizumab vs. paclitaxel + carboplatin + bevacizumab followed by maintenance bevacizumab	OS:12.6 vs. 13.4 months HR 1.00 (*p* = 0.949) PFS 6.0 vs. 5.6 months HR 0.83, *p* = 0.012
Galetta, 2015 [13]	ERACLE	Maintenance Advanced NSCLC	118	Cisplatin + pemetrexed followed by maintenance pemetrexed vs. paclitaxel + carboplatin and bevacizumab followed by maintenance bevacizumab	QoL HR 0.137, *p* = 0.078 HR 0.97, *p* = 0.41

NSCLC: non-small cell lung cancer; OS: overall survival; PFS: progression-free survival; QoL: quality of life.

**Table 2:** Randomized phase III clinical studies with VEGF receptor (VEGFR) tyrosine kinase inhibitors (TKIs) and VEGF-trap in NSCLC.

Agent	Trial	Setting	Pts	Systemic Treatment	Results
**Vandetanib**	ZODIAC Herbst, 2010 [28]	2nd line Advanced NSCLC	1391	Vandetanib + docetaxel vs. Docetaxel	PFS: 4 vs. 3.2 months HR 0.79, *p* < 0.0001 RR%: 17 vs. 10, *p* < 0.001 OS HR 0.91, *p* = 0.196
	ZEAL de Boer, 2011 [29]	2nd line Advanced NSCLC	534	Vendetanib + pemetrexed vs. Pemetrexed	PFS 0.86, *p* = 0.108 OS HR 0.86, *p* = 0.219, *p* < 0.001
	ZEST Natale, 2011 [30]	≥2nd line Advanced NSCLC	1240	Vandetanib vs. erlotinib	PFS: HR 0.98 *p* = 0.721 OS HR 1.01, *p* = 0.830
	ZEPHYR Lee, 2012 [31]	≥2nd line (prior EGFR TKI) Advanced NSCLC	924	Vandetanib vs. placebo	OS: 8.5 vs. 7.8 months HR 0.95, *p* = 0.527
**Sorafenib**	ESCAPE Scagliotti 2010 [32]	1st line Advanced NSCLC	926	Sorafenib + carboplatin/paclitaxel vs. carboplatin/paclitaxel	OS: 10.7 vs. 10.6 months HR 1.15, *p* = 0.915
	NEXUS Paz-ares, 2012 [33]	1st line Advanced NSCLC	904	Gemcitabine + cisplatin with Or without sorafenib	OS： 12.4 vs. 12.5 months HR 0.98, *p* = 0.401 PFS: 6.0 vs. 5.5 months HR 0.83, *p* = 0.008
	MISSION Paz-ares, 2015 [34]	3rd or 4th line Advanced NSCLC	703	Sorafenib vs. placebo	OS： 8.2 vs. 8.3 months HR 0.99, *p* = 0.4687 PFS: 2.8 vs. 1.4 months; HR 0.61, *p* < 0.0001
**Sunitinib**	SUN1087 Scagliotti 2012 [35]	Advanced refractory NSCLC	956	Sunitinib + erlotinib vs. erlotinib	OS: 9 vs. 8.5 months HR 0.922, *p* = 0.1388 PFS: 3.6 vs. 2 months HR 0.807, *p* = 0.0023 RR%: 10.6 vs. 6.9
**Cediranib**	BR29 Laurie, 2014 [36]	1st line Advanced NSCLC	306	Cediranib + carboplatin/paclitaxel vs. carboplatin/paclitaxel	OS 0.94, *p* = 0.72 RR%: 52 vs. 34, *p* = 0.001
**Motesanib**	MONET-1 Scagliotti JCO 2012 [37]	1st line Advanced NSCLC nonsquamous	1090	Motesanib + carboplatin/paclitaxel vs. carboplatin/paclitaxel	OS: 13 vs. 11 months HR 0.90, *p* = 0.14 PFS 5.6 vs. 5.4 months, *p* < 0.001 RR%: 40 vs. 26, *p* < 0.001
**Aflibercept**	VITAL Ramlau, 2012 [38]	2nd line Advanced NSCLC, any histology	913	Aflibercept + docetaxel vs. docetaxel	OS: 10.1 vs. 10.4 months, HR 1.01, *p* = 0.8985 PFS: 5.2 vs. 4.1 months, HR 0.82, *p* = 0.0035 RR%: 23.3 vs. 8.9, *p* < 0.001

**Table 3:** Randomized phase III clinical studies with nintedanib and ramucirumab in NSCLC.

Agent	Trial	Setting	Pts	Systemic Treatment	Results
**Nintedanib**	LUME- Lung-1 Reck, 2014 [48]	2nd line Advanced NSCLC any histology	1314	Docetaxel + nintedanib vs. docetaxel	RR%: 4.7 vs. 3.6 DCR%: 60.2 vs. 44, *p* < 0.0001 PFS: 3.4 vs. 2.7 months, HR 0.79, *p* = 0.0019 OS:10.1 vs. 9.1 months *, HR 0.94, *p* = 0.27
**Nintedanib**	LUME- Lung-2 Hanna, 2013 [49]	2nd line Advanced NSCLC Non-squamous histology	713	Docetaxel + nintedanib vs. docetaxel	RR%: 9.1 vs. 8.3 DCR%: 60.9 vs. 53.3, *p* = 0.039 PFS: 4.4 vs. 3.6 months, HR: 0.83, *p* = 0.04 OS:12.2 vs. 12.7 months, HR: 1.03, *p* = 0.79
**Ramucirumab**	REVEL Garon, 2014 [50]	2nd line Advanced NSCLC, any histology	1253	Ramucirumab + docetaxel vs. docetaxel	OS: 10.5 vs. 9.1 months HR 0.86, *p* = 0.023 PFS: 4.5 vs. 3.0 months HR 0.76, *p* < 0.0001

* OS not statistically different for all histology, but for the subgroup non-squamous histology, OS is 12.

**Table 4:** Efficacy results from main phase III clinical studies with angiogenesis inhibitors in NSCLC.

Agent	Trial	Author	Setting	Experimental Treatment	Results	Approval
**Bevacizumab**	E4599	Sandler, 2006 [8]	1st line, advanced non-squamous NSCLC	Bevacizumab + carboplatin and paclitaxel	Improvement in OS and PFS	1st line, advanced, non-squamous NSCLC
**Nintedanib**	LUME- Lung-1	Reck, 2014 [48]	2nd line, advanced NSCLC, any histology	Nintedanib + docetaxel	Improvement in PFS (any histology) and OS (non-squamous histology)	2nd line, advanced non-squamous NSCLC
**Ramucirumab**	REVEL	Garon, 2014 [50]	2nd line, advanced NSCLC, any histology	Ramucirumab + docetaxel	Improvement in OS and PFS	2nd line, advanced NSCLC, any histology
